# Development of an off-grid solar photovoltaic thermoelectric cooling system for sustainable fish preservation

**DOI:** 10.1038/s41598-026-61443-4

**Published:** 2026-07-18

**Authors:** Islam El-Sebaee, Helal Samy Helal, Abeer Wagdy Elhaddad, Ayman Ibrahim

**Affiliations:** Agricultural Engineering Research Institute (AEnRI), Agricultural Research Center (ARC), Dokki, Giza, Egypt

**Keywords:** Fish cooling, Solar energy, Thermoelectric, Monte carlo simulations, Cost analysis, Payback period, Energy science and technology, Engineering, Environmental sciences

## Abstract

Keeping fish refrigerated during the fishing process significantly enhances the quality and safety of the fish. This process is highly reliant on traditional and industrial methods, most of which are costly for small-scale fishermen, have reduced effectiveness for some, and depend on fossil fuels, hence generating GHG emissions. Therefore, a shift to controlled clean solar energy is necessary due to its feasibility and potential to reduce GHG emissions. Therefore, it aimed to assess solar energy (SE) use to develop a solar cooling unit (SCU) based on the thermoelectric cooling prototype powered by a solar photovoltaic system for fish preservation during the fishing process. Cooling chamber, heat exchanger, Peltier Modules (PMs), solar power unit, and control unit are considered the main components of the designed SCU. The results indicated that the greatest temperature reduction inside the SCU, 4 ᵒC, was achieved using a triple PM configuration at the highest specific energy consumption with a value of 0.079 kWh/kg, and a minimum performance cooling coefficient with a value of 0.039. Also, the relative cooling performance improvement values were 67 and 108% for dual and triple PM, compared to the single PM, according to the Monte Carlo statistical analysis. Moreover, the economic feasibility study indicators showed that the triple PM attached with the developed SCU is the most economically advantageous, enhancing the shortest payback period of 0.15 years. Furthermore, the robust Monte Carlo simulation revealed that the triple-PM setup provides a 76.2% probability of successfully maintaining critical preservation temperatures (T_cool_≤ 7ᵒC) under dynamic field conditions. The findings show that the thermoelectric module has a high potential in improving a direct cooling method. However, future adoption of this cooling system lies critically on new, better conversion efficiency thermoelectric material discoveries and development of higher efficiency systems.

## Introduction

Egypt covers an area of 1,001,450 km^2^, of which 6000 km^2^ is water. It is in the northeast corner of the African continent, and it borders the Mediterranean Sea and the Red Sea. It has a 2450 km coastline along both the Mediterranean and Red Seas. It possesses the richest fresh water in the region, and a unique location bordering two seas and the Nile River; hence, the fisheries form an important part of Egypt’s culture and society. Therefore, Egypt has known the profession of fishing since the dawn of history, and fishing is still practiced as an important activity^1–4^. According to^5–7^, Egypt was the sixth-largest global aquaculture producer in 2022, the third globally in tilapia production, and the top aquaculture producer in Africa. The total Egyptian aquaculture production is more than 1.6 million metric tons and valued at USD 3.5 billion as national income. The fish farming accounts for roughly 80% of the total production, and the rest total production 20% is from Capture Fisheries (Fishermen’s Catches) from natural resources such as seas, lakes, and rivers^5,7–9^. Preserving fish caught by fishermen in a cool, low-temperature environment to maintain their quality and keep them in optimal condition, a process known as fish 1st grade, is one of the most important issues facing fishermen. This cooling method of preserving fish quality plays a crucial role because it determines the selling price and increases fishermen’s income. There are diverse preserving techniques at low temperature, such as cooling, freezing, icing, and super-chilling, that are used to improve the preservation quality of fish^10–12^. To ensure that this chilling treatment preserves the quality of the fish caught within the aquatic environment, and before arriving on land. Most fishermen typically use ice to create a cold storage facility installed in the fish storage room. This ensures the fish remain in their natural state and of high quality upon their return to the mainland after some periods at sea^13^. Several studies pointed out that the fundamental purpose of cooling fish is to slow down bacterial metabolism, then prevent bacterial growth. The weight, size, and cost of ice are major obstacles to transporting large quantities of it on fishing vessels, potentially reducing the efficiency of this method and consequently lowering the quality of the fish caught. As a result, traditional fishermen resort to adding salt to the ice to overcome the limitations of the traditional ice-block cooling method and thus preserve the fish for as long as possible. This modification of the traditional ice-based cooling method can alter the taste of the fish, making it saltier^14–18^. Traditional methods, such as the use of ice blocks, present limitations as a slow cooling rate, physical damage, uneven temperature distribution, storage and handling issues, where ice blocks occupy a large storage space and need to be broken manually on board the ship, and short-term preservation^19,20^. On the other hand, there are industrial cooling systems, while effective, that present challenges such as consuming a lot of energy and leaving behind greenhouse gas emissions from refrigerants, and the consumption of energy and water resources. In addition, these industrial cooling systems are often expensive, making them unsuitable for individual small-scale fishermen^21–28^. Therefore, research and development focus on developing effective, sustainable environmental solutions for fish cooling during fishing, particularly suitable for small-scale fishermen and applicable to various fishing industry applications. Reducing greenhouse gas emissions, ensuring ease of handling and operation of a safe cooling method, and minimizing reliance on traditional energy sources. Consequently, the trend is shifting towards using solar energy to achieve this goal of a sustainable, environmentally friendly cooling method suitable for small-scale fishermen. In this regard, the International Renewable Energy Agency^29^ stated that replacing non-renewable fuel sources with renewable energy sources is the best option for improving food sustainability and addressing climate change. Furthermore^30^, mentioned that the challenge lies in transforming the food and energy sectors in a fair and environmentally sustainable manner while maintaining food security. Moreover, Egypt possesses enormous potential in the field of renewable energy, particularly solar energy, due to its geographical location and natural conditions characterized by high levels of solar irradiance. In a study on the application of solar energy in Egypt, both^31–34^ have been utilizing solar energy and the intensity of solar radiation, converting it into electrical energy through solar panels for various purposes, such as water desalination in remote and coastal areas, fish drying, a knapsack sprayer powered by a photovoltaic panel, and a house incubator unit powered by solar energy respectively. A case study from the United States in the fisheries and aquaculture sector projects that renewable energy use for the catfish sector could be as high as 41% of total direct energy use by 2050, which would result in 86% lower CO_2_ emissions but 34% higher electricity costs^22^. In the context of implementing solar-powered cooling systems^35^, a mini cooler was developed based on a solar-powered vapor compression refrigeration system for fish cooling during fishing to improve the quality and extend the shelf life of the fish catch. The results showed that the vapor compression refrigeration system powered by solar energy operated optimally, with system performance indicated by a COP value of 2.24–2.42. Many studies about the thermoelectric module (TEM )generator’s applications demonstrate significant progress; several challenges persist, for example, low efficiency, thermal conductivity, and electrical resistivity. Also, studies focusing on material optimization pointed out that the advances in nanostructured and high-ZT materials could help overcome these limitations of the thermoelectric generators. In this regard^36,37^, concluded that the TEM generators present a promising pathway for the efficient utilization of industrial waste heat and renewable thermal energy sources. In the context of searching for simple, low-cost cooling methods. Moreover^38–40^, stated that the TEM is a solid-state device that operates on the principle of the Peltier effect, converting heat into electricity and vice versa. In addition, it is used as a micro-generator for power generation or as a micro-refrigerator for cooling applications. The TEM does not use any refrigerants for cooling and thus presents an alternate, clean cooling technology, leaving behind no chemical residues. Also^36^, pointed out that the developed TEM, which does not rely on refrigerants, offering portability, as well as stable temperature control, has an appropriate option for cooling in off-grid areas. In this context^41^, designed a TEM prototype based on the Peltier effect, aimed at remote areas. This is to maintain a specified temperature, perform temperature control in the range of 5 to 25 °C, and provide refrigeration. As well^42^, evaluated the implementation viability of the TEM system powered by solar energy on a large scale. The findings suggest that the prototype could lead to a qualitative leap in industrial refrigeration systems by providing a sustainable, cost-effective, and eco-friendly alternative to traditional refrigeration systems. Furthermore^14^, designed and manufactured a cool box of fish storage composed of solar photovoltaic, a charge controller, Arduino UNO, and a shock device based on solar energy. The findings point out that the solar panel produced an average of 2.53 kW of energy in a day, which can power the cool box device for 10 h. Also, the temperature control helps the tool save energy, estimated at 170 min longer than the cooling unit without control. Additionally^43^, investigated the simultaneous cooling and heating using a TEM to simultaneously cool and heat water. It finds that 1 L of water was cooled from 40 to 16 ᵒC in 60 min, and heated from 25 to 45 ᵒC. As a result, the energy consumption was about 0.83 kWh with a 1.33 coefficient of performance. Similarly^44^, fabricated a TEM powered by solar energy and evaluated its cooling potential for fish preservation. Finds that within 90 min., the temperature of the TEM decreased to 7.4 ᵒC and then reached 5 ᵒC in 150 min. Moreover, the TEM achieved a 0.44 coefficient of performance. In addition^45^, studied a techno-economic analysis of a walk-in simulation in a cold fish storage room powered by a solar PV mini-grid. The results indicated that it is economically feasible to implement a communal cold storage unit if an investor makes the initial investment and allows the fishers at least a pay-back period. Following the same methodology^46^, investigated a TEM with different numbers of Peltier units (1 to 4) and different refrigerants on thermal progress performance in terms of thermal absorption and energy consumption. The results showed that the mixture (distilled water + mono ethylene glycol 50:50) outperformed distilled water only, achieved faster temperature reduction, and a higher COP, with a value of 3.75 at 4 Peltier units, with a mixture of mono ethylene glycol. Based on the above, this study aims to provide an environmentally friendly cooling system suitable for small-scale fishermen during fishing. This will be achieved by designing a cooling unit within the range of suitable cooling temperatures for fish, from 4 to 8 ᵒC., that utilizes thermoelectric cooling powered by solar energy. Furthermore, a feasibility study for disseminating this technology to small-scale fishermen.

While the absolute target is maintaining ≤ 4 °C, dynamic solar irradiance and harsh field conditions mean that absolute thermal stability is not guaranteed 100% of the time. Therefore, the realistic design objective is to maintain the catch within the acceptable safety threshold of 4 to 8 °C during the fishing operation.

### Materials and methods

A series of experiments was conducted in this study to implement, test, and evaluate the automatic solar cooling unit (SCU) developed at the Testing and Research Station for Tractor and Farm Machinery in Sabahia, Alexandria, Egypt, with a latitude of 31.23341 N and longitude of 29.97559E, Agricultural Engineering Research Institute (AEnRI), Agricultural Research Center (ARC). Twenty kilograms of fresh fish for each batch were collected as a raw material for testing and evaluating the developed SCU from the fresh fish market in Bakos, Alexandria, Egypt, during the period spanning from 5 to 13 August 2024. The working environment was also monitored in terms of temperature, humidity, and wind intensity using an environmental meter (model: EM-9300SD).

### Solar cooling unit developed

**Fig. 1:**
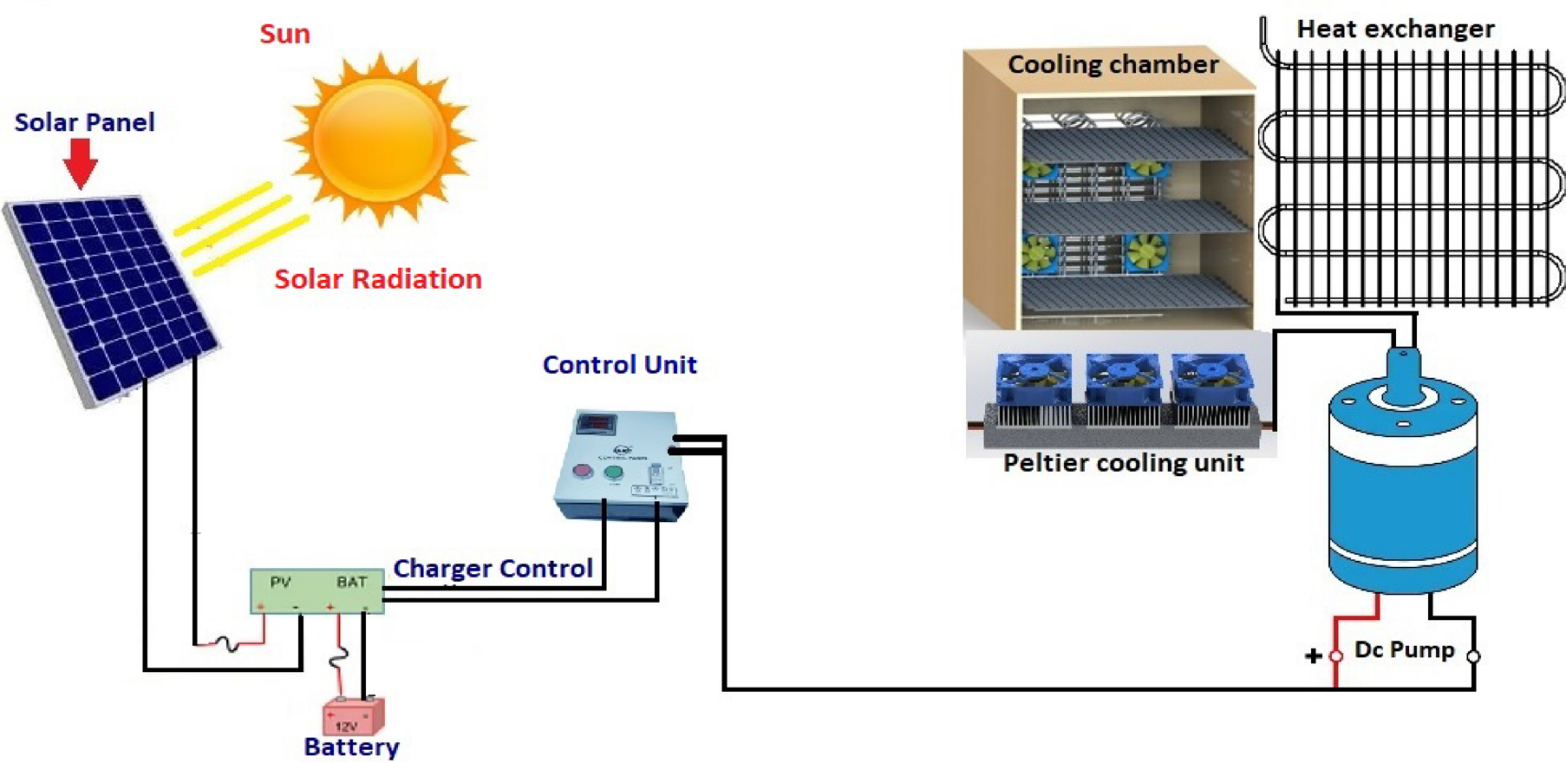
Illustrating the main components of the developed SCU.

The basic concept of the developed SCU under study is based on harnessing solar energy and converting it into electrical energy, in addition to an automated control unit to operate the SCU efficiently and reduce energy consumption through intelligent self-regulation. This makes it suitable for small-scale fishermen during fishing operations. The developed SCU consists of five main parts: a cooling chamber, a heat exchanger, Peltier units, a solar power unit, and a control unit (Fig. 1). A detailed description of the developed SCU is as follows.

#### Cooling chamber

**Fig. 2:**
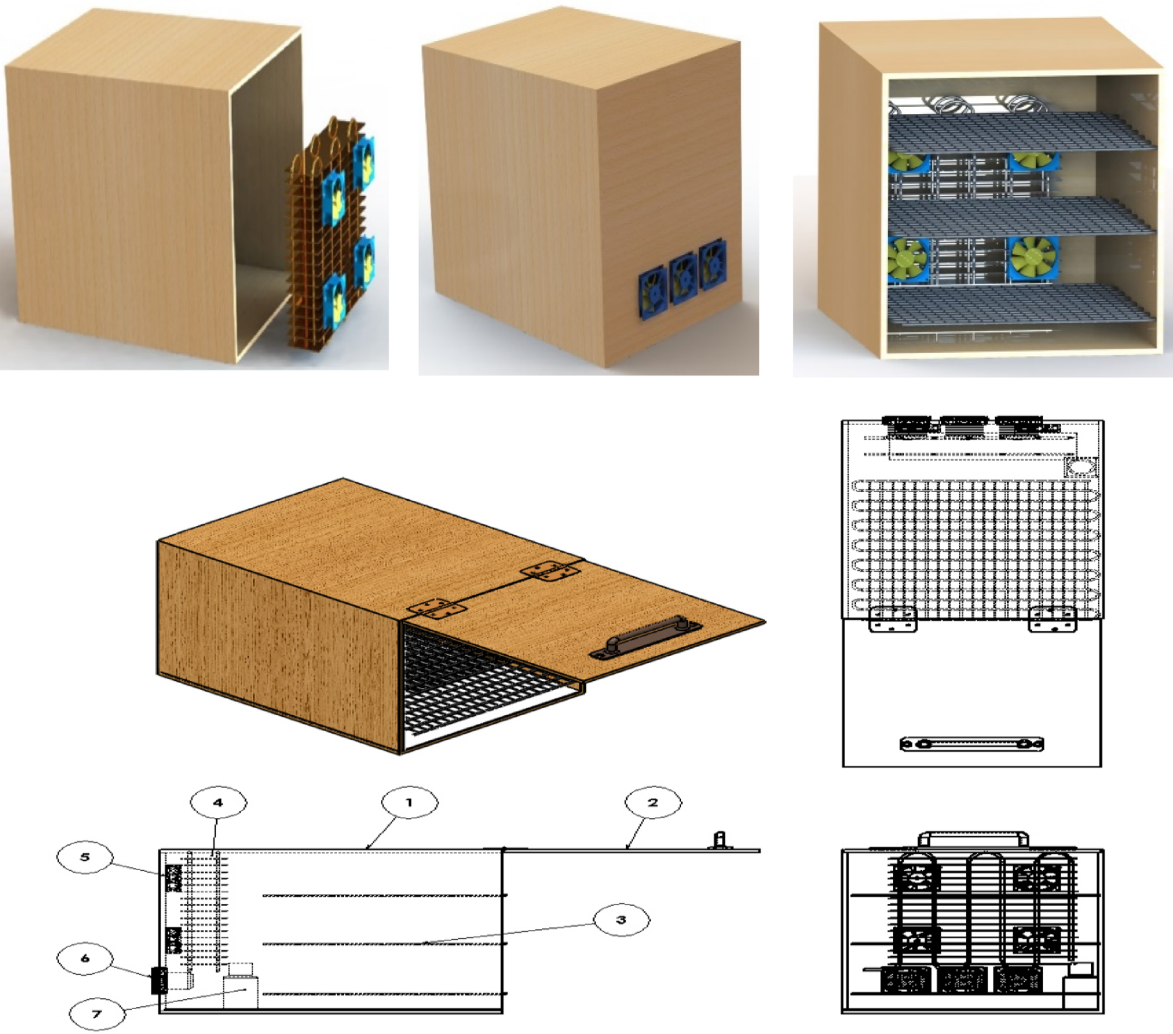

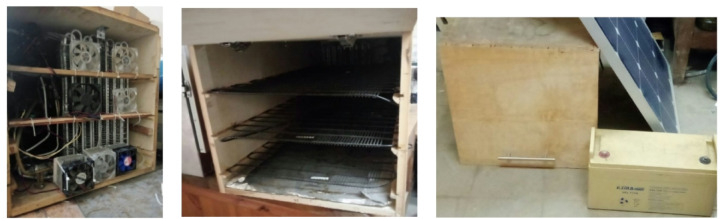
Illustrates the cooling chamber components of the SCU. (1) Cooling chamber, (2) SCU door, (3) Shelf, (4) Heat exchanger, (5) Fans, (6) PM on the water tank surface with heat sinks, (7) DC pump.

The cooling chamber is the central component of the SCU. It is constructed of 18 mm thick plywood and is rectangular in shape, with dimensions 500 × 600 mm, and 500 mm height (Fig. 2**)**. It contains three galvanized aluminum shelves, each with dimensions 500 × 450 mm, with a 1 mm hole perforated metal mesh. The first shelf was positioned 50 mm above the base chamber, then a 100 mm gap was maintained between successive shelves.

Additionally, four fans (12 V–0.1 A − 2200 rpm, 37.7 CFM, and 2.23 mmHg) were installed at the back part of the cooling chamber. This was done to ensure the uniform distribution of the convection of the cold air inside the cooling chamber.

#### Heat exchanger

**Fig. 3:**
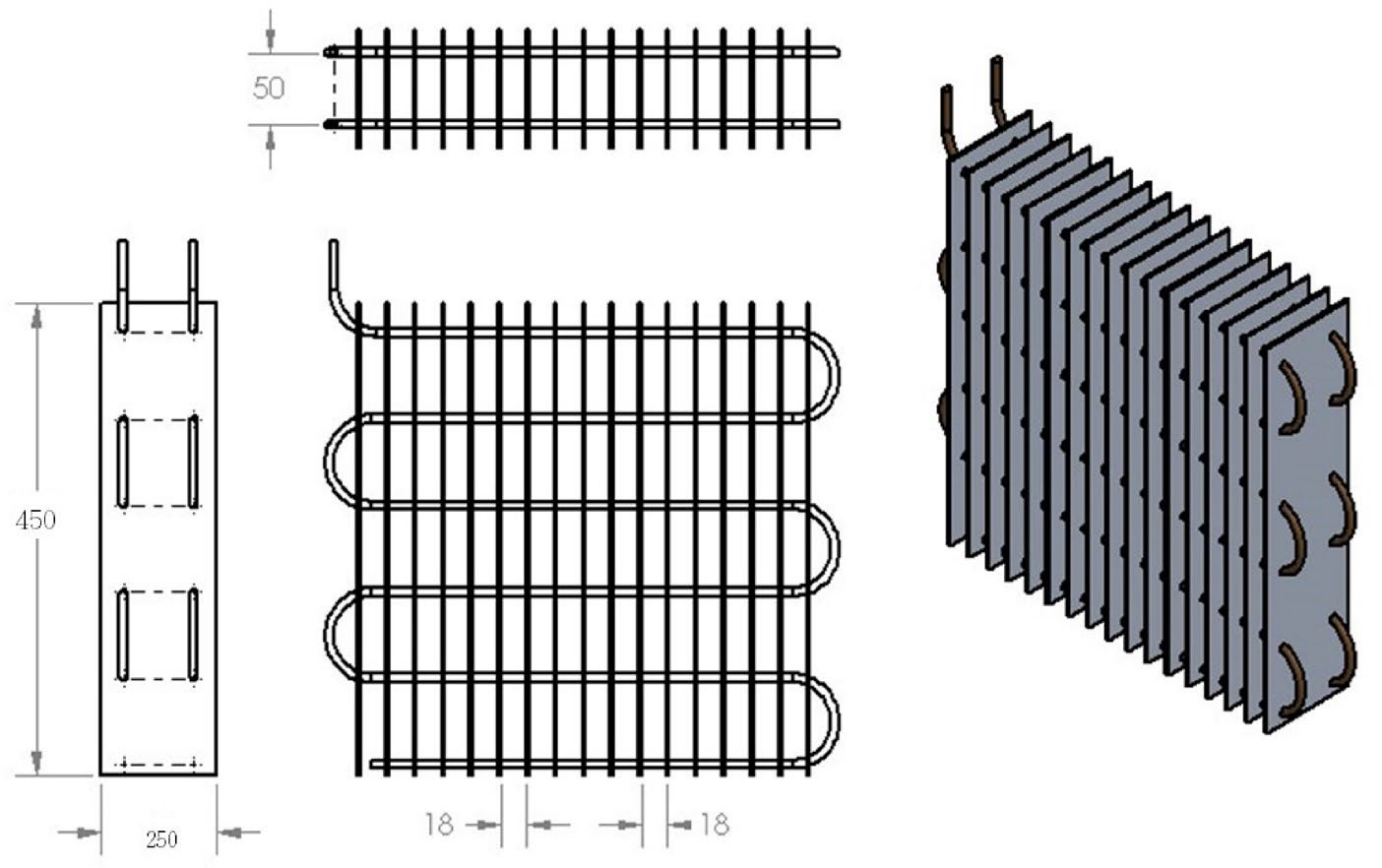
Demonstrates the heat exchanger showing aluminum tubes and fins.

The purpose of the heat exchanger is to absorb heat within the cooling chamber and transfer it using water as the medium inside the heat exchanger tubes. The dimensions of the heat exchanger were 450 × 250 × 50 mm, with length, width, and height, respectively, as shown in Fig. 3. It is mounted in front of the air distribution fans, using an 8000 mm long aluminum tube with a vertical diameter of 6 mm, positioned behind the chiller to ensure efficient water flow. In addition, Table 1 highlights a detailed technical description of the shape and materials of the heat exchanger^47–50^.

**Table 1:** Technical specifications of the custom-fabricated fin-and-tube heat exchanger.

Parameter	Symbol	Value	Unit	Measured / Calculated	Formula / Basis	Reference
Overall length	L	450	mm	Measured	Direct measurement	—
Overall width	W	250	mm	Measured	Direct measurement	—
Overall depth	D	50	mm	Measured	Direct measurement	—
Material	—	Aluminum	—	Observed	Visual inspection + fabrication record	—
Heat exchanger type	—	Fin-and-tube, serpentine	—	Observed	Visual inspection	—
Number of fins	Nf	17	—	Measured	Direct count	—
Fin pitch	fp	3.1	mm	Calculated	fp = D / (Nf − 1) = 50 / 16 = 3.1 mm	^47^
Fin thickness (estimated)	δf	≈ 0.3	mm	Estimated	Typical value for aluminum plate fins	^48^
Tube outer diameter	do	6	mm	Measured	Direct measurement (caliper)	—
Tube inner diameter	di	5	mm	Calculated	di = do − 2t = 6 − 2(0.5) = 5 mm (wall t ≈ 0.5 mm, standard Al tube)	^50^ (Al drawn tube)
U-bends (one side)	Nu	6	—	Measured	Direct count	—
Number of tube passes	Np	12	—	Calculated	Np = 2 × Nu = 2 × 6 = 12	^47^
Tube vertical spacing	St	37.5	mm	Calculated	St = L / Np = 450 / 12 = 37.5 mm	^47^
Total tube length	Ltube	≈ 8,000	mm	Calculated	Ltube = 2 rows × Np × W + U-bend lengths = 2 × 12 × 250 + ~ 2000 ≈ 8,000 mm	Manufacturer fabrication record
Fin heat transfer area	Afin	3.82	m²	Calculated	Afin = Nf × 2 × L × W = 17 × 2 × 0.45 × 0.25	^49^
Tube bare area	Atube	0.03	m²	Calculated	Atube = π × do × Ltube × (fin gap ratio)	^48^
Total heat transfer area	Atotal	≈ 3.85	m²	Calculated	Atotal = Afin + Atube	^49^

Measured: direct physical measurement; Calculated: derived from measured values using established equations; Estimated: typical value from literature; Observed: visual inspection/fabrication record.

#### Peltier cooling unit

**Fig. 4:**
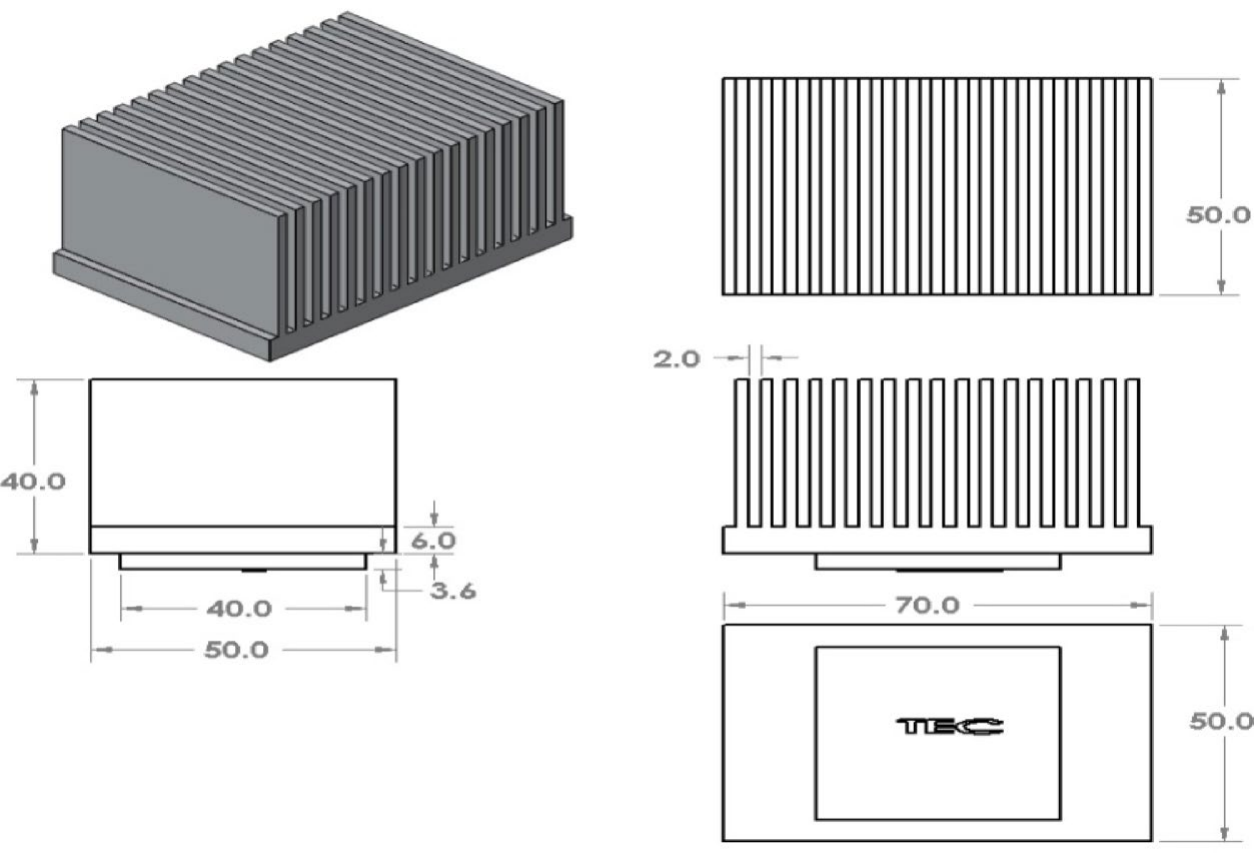
Illustrates the PM structure.

The Peltier cooling unit is comprised of a water tank (300 × 40 × 33 mm length, width, and thickness, respectively) and a Peltier module (PM)(TEC1-12706) (Rated voltage,12 V DC, Vmax = 15 V) (Working current: 3–4 A(rated 12 V); Imax: 6 A (Operating temperature: -30 to 70 ^o^ C) ( Maximum power 60 Watts) with dimensions 40 × 40 × 4 mm length, width, and thickness as shown in Fig. 4.

**Fig. 5:**
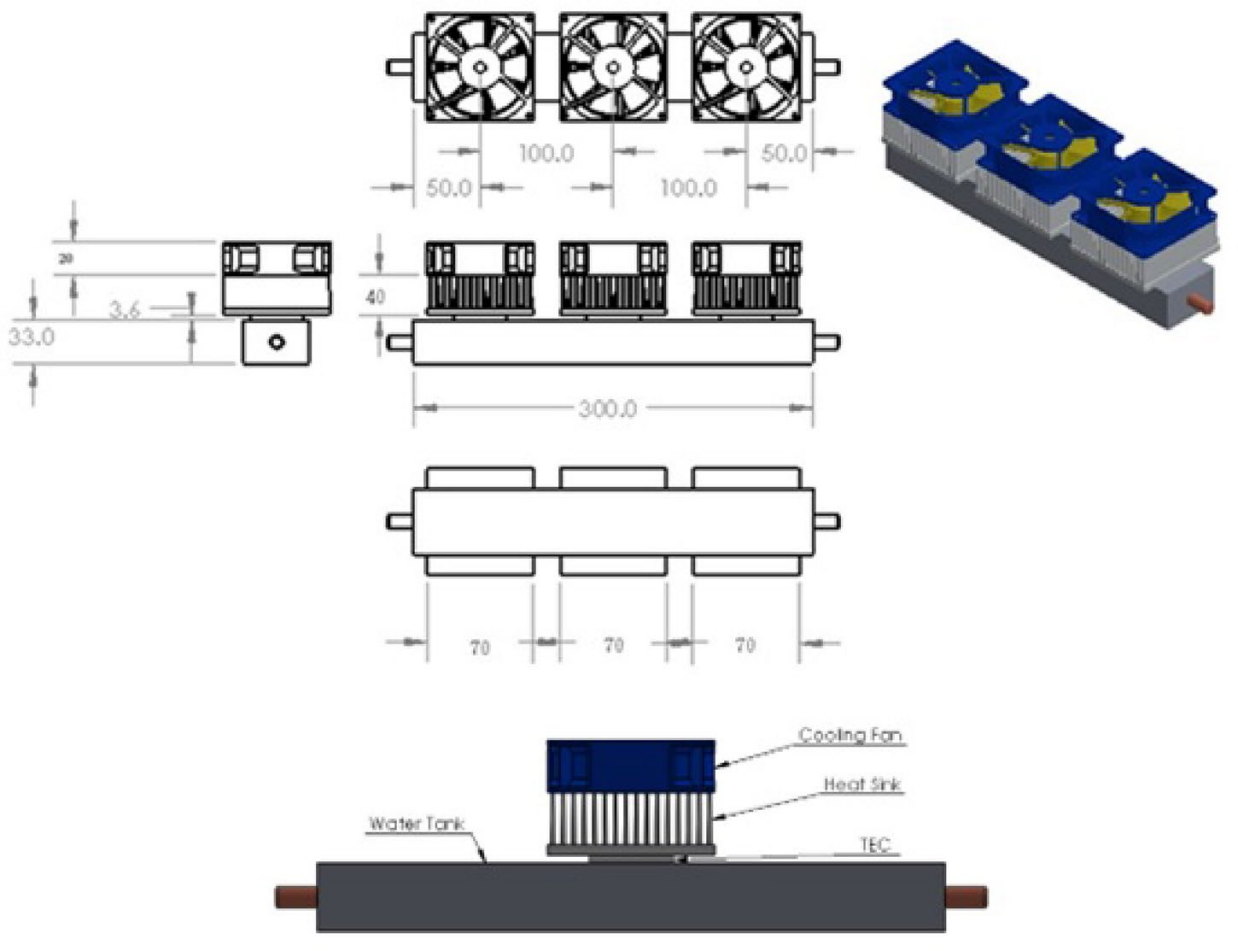
PM dimensions and distribution method on the surface of the water tank inside the cooling chamber.

The PM core components include an array of semiconductor pellets (P/N Pairs), electrically connected in series but thermally in parallel, all sandwiched between two highly conductive ceramic plates, one of which becomes a heat absorption (cold side) and the other a heat rejection (hot side). The hot side of the PM is attached to the Aluminum heat sink with thermal conductivity (204.3 W/m °C), and emissive (0.02–0.9) at 25 °C to ensure continuous cooling. The dimensions of the heat sink were 40 × 50 × 70 mm height, length, and width, with a 6 mm base thickness. It contains 22 fins from Alumina (Al (6063-T6), 1.2 mm in thickness, and the spacing between fins is 2 mm with forced convection heat transfer mode. As shown in Fig. 5, three PMs were installed on the top of the water tank in the back part of the cooling chamber, the first PM was installed at a 50 mm height from the chamber base and on top of the water tank, and a 100 mm distance was left between each PM.

#### Solar power unit

The solar power unit was composed of a solar panel with dimensions of 1504 × 1002 × 35 mm in length, width, and height. The maximum power output of this panel is 300 W, with a rated voltage and current of 32 V and 9.4 A. It connected with an MPPT solar charge controller (50 A-12 V/24V/48V, PWM solar panel smart battery regulator, LCD solar panel regulator for RV, Photovoltaic Systems) to charge a 12-100 A lead acid battery. Once the battery was fully charged, it immediately supplied the electrical current to start the PM, fans, and a D.C. pump (12 V, 15 W) with a 0.67 × 10^− 3^ kg/sec flow rate. This pump is responsible for circulating water from the cooler tank under the PM through the cooler exchanger and subsequently into the tank. The solar panel was strategically oriented towards the south with a constant tilt angle of 31°, and the testing period spanned from 5 to 13 August 2024. Solar radiation intensity was recorded in half-hour intervals using a solar power meter (model: SPM-1116SD).

#### Control unit

**Fig. 6:**
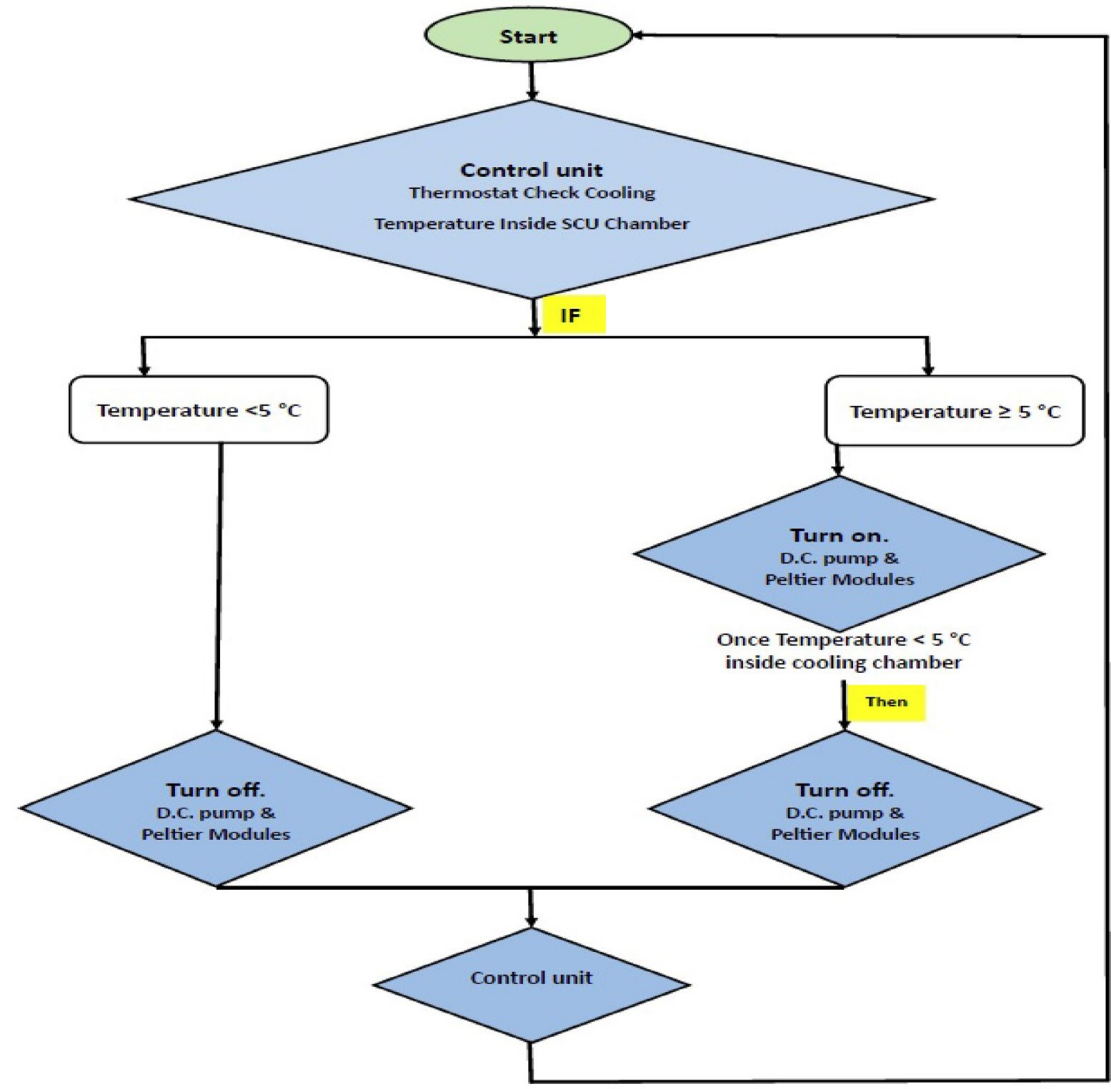
Highlights the flowchart of the steps involved in the SCU control process.

The control unit was designed to monitor and maintain the temperature inside the SCU within the specified range for the cooling process (5 °C), as shown in the flowchart Fig. 6. It consists of alloy steel thermostats (TR2 9934 type, Italy) with a temperature range of -10 to 110 °C and an accuracy of ± 1 °C. These thermostats are set at 5 °C inside the cooling chamber and water tank. The control unit is also equipped with temperature sensors to continuously monitor the cooling temperature inside the SCU and take action. Furthermore, the thermostat monitors the temperature inside the cooling chamber. If the temperature inside the chamber rises above the optimum temperature (5 °C), the thermostat sends a signal to activate the water pump, which immediately circulates chilled water into the heat exchanger to restore the temperature to the permissible level. Once the permissible cooling temperature is reached, the thermostat sends a signal to cut off the power supply and stop the pump. In addition, there are two sensors (K-type 0 to 70 °C) used to measure the ambient temperature and the temperature in the cooling chamber.

### Uncertainty analysis

Measurement uncertainty was evaluated following the Type B methodology prescribed by the Joint Committee for Guides in Metrology^51^. Since instrument calibration data were unavailable, the half-width of each manufacturer’s stated accuracy tolerance, denoted a, was treated as the sole source of uncertainty information. In the absence of any additional distributional data, a rectangular (uniform) probability distribution was assumed for each instrument, as recommended in § 4.3.7 of the GUM for cases where the measurand is equally likely to take any value within a symmetric interval [− a,+a]^51^. Under this assumption, the Type B standard uncertainty is defined by Eq. 1.

1 $$U_{B} = \frac{a}{{\sqrt 3 }}$$

which represents the standard deviation of the rectangular distribution whose half-width is a. The factor √3 arises from the variance of a uniform distribution defined over [− a,+a], i.e., σ^2^ = a^2^/3^51^. For derived quantities involving two independent measurements, the combined standard uncertainty was obtained through standard uncertainty propagation (law of propagation of uncertainty, GUM § 5.1.2). Since the temperature difference ΔT=Tair - *T*cool is computed from two uncorrelated K-type thermocouples with equal standard uncertainties Eq. 2.

2 $$u_{c} \left( {\Delta T} \right) = \sqrt {u^{2} \left( {T_{{air}} } \right) + u^{2} \left( {T_{{cool}} } \right)} = \sqrt 2 \times u_{{B,K}}$$

Where u_B, K_ =1.5/√3 = 0.866 $^{ \circ } C$ is the standard uncertainty of a single IEC 60584-1 Class 1 thermocouple, giving u_c_(ΔT) = 1.225 $^{ \circ } C$ Finally, the expanded uncertainty was obtained by multiplying each standard uncertainty by a coverage factor k = 2, Eq. 3.

3 $$U = k \times u_{B} = 2 \times \frac{a}{{\sqrt 3 }}$$

A coverage factor of k = 2 was selected because, by the central limit theorem, the combination of multiple rectangular distributions converges toward a normal distribution, for which k = 2 corresponds to a coverage probability of approximately 95%^51,52^ . The resulting expanded uncertainties for all instruments are summarized in Table 2.

**Table 2:** Illustrates measurements uncertainty analysis, GUM methodology (Type B, K = 2).

No.	Instrument	Measured Quantity	Operating Range	Resolution	Manufacturer Accuracy	Standard Uncertainty U_B_ (°C or unit)	Expanded Uncertainty U k = 2	Source
1	PCE-SPM1 (Solar meter)	Solar irradiance (W/m²)	0–2000 W/m²	1 W/m²	± 10 W/m² or ± 5% (higher applies)	17.32 W/m²	± 34.6 W/m² (± 5.8%)	PCE Instruments official datasheet ✓
2	Lutron (Tair)	Air temperature (°C)	0–50 °C	0.1 °C	± 0.8 °C	0.462	± 0.92 °C	Lutron official PDF datasheet ✓
3	Lutron (Humidity)	Relative humidity (%RH)	0–95%RH	0.1%RH	± 3% RH (< 70%RH) ±(3%rdg + 1%) (≥ 70%)	1.732	± 3.46%RH	Lutron official PDF datasheet ✓
4	Lutron (Air velocity)	Air velocity (m/s)	0.4–25 m/s	0.1 m/s	±(2%rdg + 0.2 m/s) @ 2.0 m/s: ±0.24 m/s	0.139	± 0.277 m/s	Lutron official PDF datasheet ✓
5	Lutron + K-probe	Temperature via probe (°C)	-50 to 1300 °C	0.1 °C	±(0.4%rdg + 0.8 °C) @ 30.0 °C: ±0.92 °C	0.531	± 1.06 °C	Lutron official PDF datasheet ✓
6	K-type thermocouple	Tair & Tcool (°C)	0–70 °C	—	± 1.5 °C (Class 1, T < 375 °C)	0.866	± 1.73 °C	IEC 60584-1 standard ✓
7	TR2 9934 thermostat	Control temperature (°C)	-10 to 110 °C	—	± 1.0 °C (typical, not in datasheet)	0.577	± 1.15 °C	IMIT Italy website (accuracy not listed)

### Total and specific energy consumption

The energy consumption by the cooling system for cooling fresh fish was calculated according to the cooling system used for the automatic SCU. The energy consumption of the Peltier Module, push air fans inside the cooling chamber, and the water pump was calculated according to Eq. 4, according to^34,53^.

4 $$\:TEC_{{SCU}} = \left( {P_{{PM}} \times \:t_{{PM}} } \right) + \left( {P_{{PF}} \times \:t_{{PF}} } \right) + \left( {P_{{WP}} \times \:t_{{WP}} } \right)$$

Where, $\:{P}_{PM}$, $\:{P}_{pF}$, and $\:{P}_{WP}$, the power required of the Peltier Module, push air fans inside the cooling chamber, and the water pump, respectively, in watts (kW). Also, the $\:{t}_{PM}$, $\:{t}_{PF}$, and $\:{t}_{WP}$, are the cooling times (h) over which the energy was consumed for the Peltier Module, push fans, and the water pump for the SCU, respectively. Hence, the $\:{TEC}_{SCU}$, is the calculated total energy consumed (kWh). Then, the specific energy consumption (SEC) to cool 1 kg of fresh fish was calculated according to Eq. 5^53^.

5 $$\:SEC = \:\frac{{TEC_{{SCU}} }}{{Q_{{Fish}} }}$$

Where $\:{Q}_{Fish}$, is the fresh fish mass (kg), and the SEC is the specific energy consumption (kWh/kg). Also, the cooling efficiency (η_scu_) (°C/W) was calculated according to Eqs. 6 & 7.

6 $$\eta _{{{\mathrm{scu}}}} = {\text{ }}\Delta {\text{T }}/TEC_{{SCU}}$$

7 $$\Delta {\mathrm{T}} = {\text{T ambient }} - {\text{T cooling chamber}}$$

Where ΔT is the temperature differential between ambient and cooling chamber temperatures, Eq. 7. Finally, the performance coefficient (*CP*_*scu*_) of the SCU was calculated according to Eqs. 8, 9, 10 & 11. Where the cooling rate by unit Joule (watt-second, 1 J=1Ws) inside the chamber of the SCU can be calculated as Eqs. 10 & 11. The C represents the specific heat of unfrozen mullet, which is approximately 3.35 to 3.77 kJ/kg·ᵒC, and t is the total time required for cooling in each cooling system (h).

8 $$\:{CP}_{SCU}=\frac{{Q}_{Cooling\:\left(kW\right)}}{{PTC}_{SCU}}$$

9 $$PTC_{{SCU}} = \left( {E_{{PM}} \times t_{{PMt}} } \right) + \left( {E_{{PF}} \times t_{{PFt}} } \right) + \left( {E_{{WP}} \times t_{{WPt}} } \right)$$

Where, $\:{E}_{PM}$, $\:{E}_{pF}$, and $\:{E}_{WP}$, are the energy consumed by the Peltier Module, push air fans inside the cooling chamber, and the water pump, respectively, in kW/h. Also, the $\:{t}_{PMt}$, $\:{t}_{PFt}$, and $\:{t}_{WPt}$, are the total cooling times (h) of the Peltier Module, push air fans inside the cooling chamber, and the water pump, respectively. Hence, the $\:{PTC}_{SCU}$, is the calculated total power (kW).

10 $${\mathrm{Q}}_{{{\text{Cooling }}({\mathrm{J}})}} = Q_{{Fish}} \times C \times \Delta T$$

11 $$Q_{{Coolin~\left( W \right)}} = \frac{{Q_{{Cooling~\left[ {J\left( {Ws} \right)} \right]}} }}{{t\left( s \right)}}$$

It is crucial to note that the calculated CP_SCU_ represents the overall System Coefficient of Performance COP_system_ based strictly on the useful sensible heat removed from the fish, rather than the isolated thermodynamic COP of the Peltier modules. The total power) T_CSCU_) includes the parasitic loads of the circulating pump and distribution fans.

### Statistical validation

Monte Carlo simulations (*N* = 10,000 iterations) were performed in Google Colab (Python 3.8; NumPy, SciPy, Matplotlib, Seaborn) to analyze and validate the experimental results of the PM cooling application. The initial transient measurement at t = 0 (where T_cool ≈ T_air) was excluded from all statistical computations, retaining *n* = 11 steady-state observations per configuration. For each configuration (single, dual, and triple PM), the temperature difference between ambient air and the cooling chamber was computed as ΔT = T_air − T_cool and characterized by its experimentally measured mean and standard deviation (µ, σ). Two independent sources of variability were explicitly modelled for each Monte Carlo iteration: (i) temporal variability in ambient and cooling conditions, represented by sampling from a Normal distribution N(µ, σ) fitted to the steady-state measurements; and (ii) instrument measurement uncertainty of each K-type thermocouple (IEC 60584-1, Class 1, ± 1.5 °C), modelled as a rectangular probability distribution U(− 1.5, + 1.5) °C following JCGM 100:2008 4.3.7, yielding a standard uncertainty u_B = 0.866 °C per sensor, sampled independently for each iteration. Within this probabilistic framework, 10,000 random realizations were generated for each case, enabling the estimation of 90% confidence intervals, coefficients of variation (CV), and the probabilities of meeting critical thermal thresholds (ΔT ≥ 20 °C, T_cool ≤ 7 °C^54,55^. The computational workflow consisted of experimental data preprocessing, temperature-difference calculation, statistical parameter extraction, Monte Carlo simulation execution, and graphical visualization, with a focus on confidence-interval estimation, probabilistic performance assessment, and temporal stability analysis.

### Cost analysis of the SCU

The following paragraph highlights the calculation of the costs of the developed SCU for preserving fish during the fishing process, and Table 3 shows the costs of the raw materials used in manufacturing the SCU.

**Table 3:** Illustrative costs of the SCU raw materials.

No.	Raw materials parts	Quantity	Total Cost ($)	Total Cost (LE)
1	Cooling chamber	1	10	476.27
2	Heat exchanger	1	6	285.76
3	DC pump	1	3	142.88
4	Solar panel	1	60	2857.60
5	Battery	1	60	2857.60
6	Charger control	1	8	381.01
7	PM cooling system	1	30	1428.80
8	Control unit	1	5	238.13
9	Total	1	182	8668.04

The approximate costs of raw materials used in manufacturing the SCU were included, based on the local raw material prices in the Egyptian market. These costs were calculated based on the exchange rate of the US dollar against the Egyptian pound, which is approximately equivalent to 1$≈47.6266 LE. Initially, the first annual cost of the SCU was calculated from Eq. 12^56,57^. Where *PC*_*SCU*_ is the principal cost of the SCU structure, and CRF is the capital recovery factor. To find the cost value of an SCU, Eq. 13 is used to calculate the CRF. The CRF was calculated based on the interest rate of the transferring banks in (IR, %), and n is the lifespan average of the SCU in years.

12 $$Cost_{{SCU}} = PC_{{SCU}} \times CRF$$

13 $$CRF = \frac{{IR\left( {1 + IR} \right)^{n} }}{{\left( {1 + IR} \right)^{{n~}} - 1}}$$

In this regard, Eq. 14 was used to calculate the Salvage annual of the SCU. The Salvage value performs the approximated value of the SCU at the end of its service life and is typically estimated at 50% of its initial cost. The SFF is the sinking fund factor, which is determined from Eq. 15^57^. The salvage value of the SCU was calculated according to Eq. 16^58^.

14 $$Salvage_{{annual}} = SFF \times Salvage_{{Value}}$$

15 $$SFF = \frac{{IR}}{{\left( {1 + IR} \right)^{n} - 1}}$$

16 $$Salvage_{{Value}} = 0.2 \times PC_{{SCU}}$$

Furthermore, the total annual cost of the SCU ($\:Annual\:{Cost}_{SCU})$ was calculated based on Eq. 17, where the annual maintenance cost (*MC*_*annual*_) was obtained from Eq. 18^57,58^.

17 $$Annual~Cost_{{SCU}} = Cost_{{SCU}} + MC_{{annual}} - Salvage_{{annual}}$$

18 $$MC_{{annual}} = 0.15 \times PC_{{SCU}}$$

The cost of chilled fish/ kilogram produced (CCF_kg_) from the SCU was obtained from Eq. 19 according to^58^. Where, $\:Avg.Productivity$ is the average annual productivity of chilled fish produced. Lastly, the payback period of the SCU was calculated based on Eq. 20, where PCF_kg_ is the price of chilled fish/ kilogram.

19 $$CCF_{{kg}} = \frac{{Annual~Cost_{{SCU}} }}{{Avg.Productivity}}$$

20 $${\mathrm{Payback~period}} = \frac{{Total~Cost_{{SCU + MC_{{annual}} }} }}{{Avg.Productivity \times PCF_{{kg}} }}$$

The economic feasibility was modeled based on the typical operational profile of artisanal coastal fishermen targeting high-value fish species. Field observations indicated an average daily catch of 3 kg per fisherman. Assuming a conservative operational schedule of 5 fishing days per week (excluding weekends, rough weather days, and holidays), the system is active for approximately 240 days annually. Consequently, the average annual productivity is estimated at 720 kg/year (3 kg/day × 240 days). Based on a unit total cost of 182 USD, an annual maintenance cost of 27.3 USD, and a chilled fish premium selling price of 2 USD/kg, the payback period was calculated to be exceptionally short at 0.15 years, confirming its viability for low-income fishermen.

## Results and discussion

**Fig. 7:**
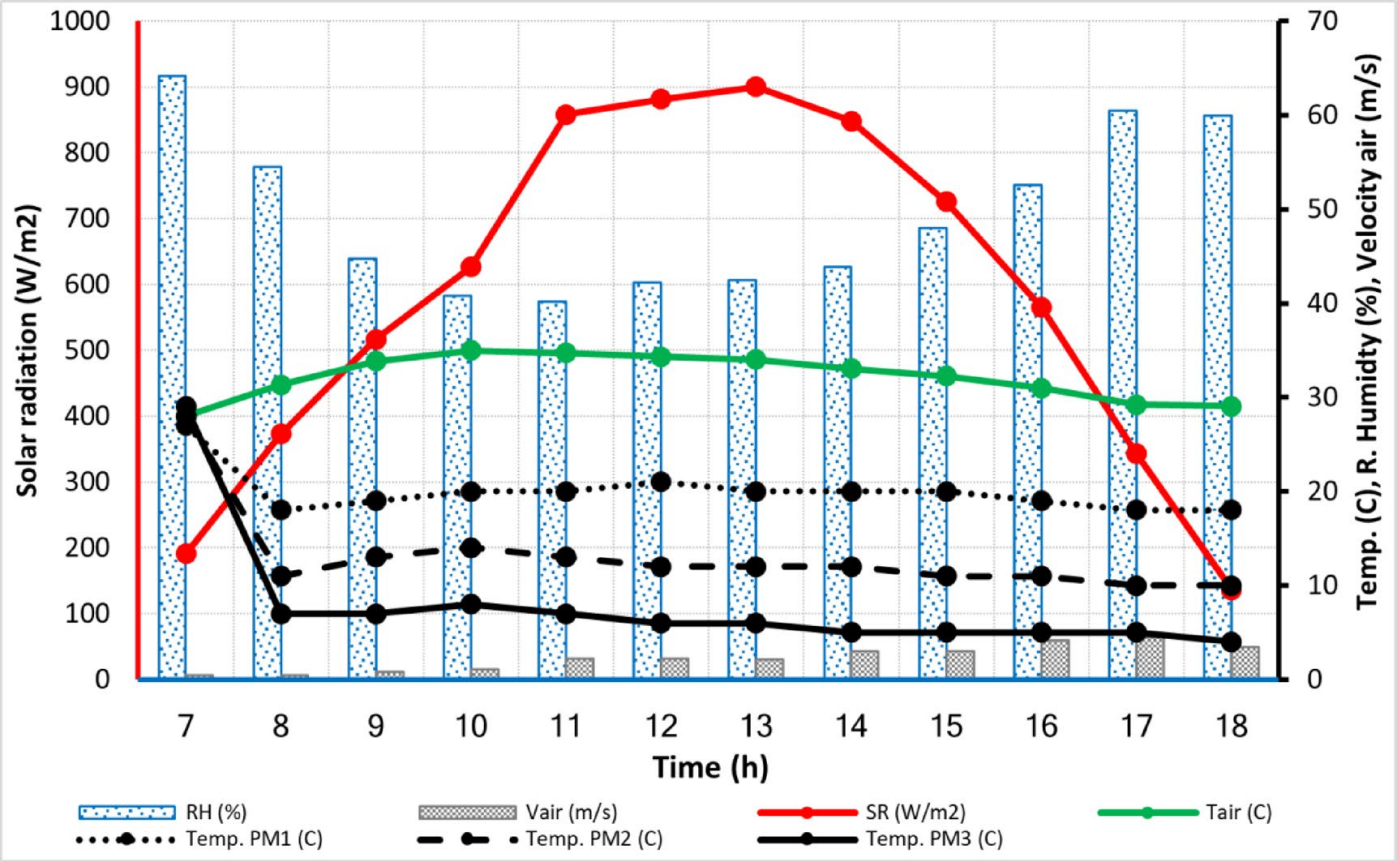
Ambient environmental conditions vs. SCU temperature using 1, 2, and 3 PM.

Studying the environmental conditions surrounding the SCU development experiment revealed a clear pattern of behavior throughout the day, as shown in Fig. 7.

Regarding solar radiation intensity, measurements showed an upward trend until peak hours, then a gradual decrease until sunset. Solar radiation increased from approximately 191–228 W/m² at 7:00 am to a peak of between 882 and 939 W/m² around peak hours, before decreasing towards the end of the afternoon, and then decreasing to values ranging from 344 to 136 W/m² towards the end of the afternoon and before sunset. As a result of the varying intensity of solar radiation throughout the day, both ambient air temperature and relative humidity were affected. The ambient air temperature curve followed the same pattern as the solar radiation intensity. The ambient air temperature rose from approximately 28–31 °C between 7:00 and 8:00 am, reaching its peak of 34–35 °C during the peak hours, before gradually decreasing towards the end of the afternoon and the beginning of sunset, reaching a low of 29 °C. In contrast to the behavior of solar radiation intensity and ambient air temperature, the relative humidity pattern was characterized by its highest values at the beginning of the day, ranging from 54 to 64% in the early morning hours. It then decreased as solar radiation intensity increased and ambient air temperature rose, reaching 40 to 42% during peak hours. From the afternoon until sunset, it began to rise again, reaching 53 to 61%. This increase is attributed to the significant decrease in solar radiation intensity and temperature during the sunset period. Meanwhile, the ambient air velocity pattern followed a semi-increasing pattern, ranging from 0.4 m/s in the morning to 4.5 m/s just before sunset. The results of measurements of solar radiation intensity behavior, along with related measurements of ambient temperature, wind speed, and relative humidity, are consistent with the findings of^31^, solar-powered desalination using on-grid-connected electricity generation^59^, passive solar-powered desalination^60^, hybrid solar drying^53^, presented a practical vision exploring reducing the carbon footprint of the drying process through solar energy applications^61^, hybrid solar dryer for drying medicinal and aromatic plants^33^, insect trap powered by off grid solar energy connected, and, house incubator unit powered by off grid solar energy connected. These environmental conditions directly impacted both the PV panel used for electricity generation and, consequently, the performance of the SCU. Under full-load field conditions, the cooling chamber was fully stacked with its maximum capacity of 20 kg of fresh fish. The thermal pull-down tests revealed that the triple-PM configuration required approximately 4 h from initial startup to overcome the initial sensible heat of the fish and achieve an average stable storage temperature of 6 °C. It was observed that increasing the number of Peltier modules (PM) resulted in a greater reduction in the temperature inside the SCU. The greatest temperature reduction inside the solar fish cooling unit was achieved when using 3 PM. The triple PM configuration achieved the greatest reduction in temperature inside the SCU, reaching 4–8 °C early and maintaining low temperatures throughout the day, demonstrating its ability to overcome both ambient heat load and irradiance fluctuations. The second and third positions were occupied by the dual and single PM cooling systems, respectively. Both achieved a significant temperature reduction inside the cooling unit and maintained it throughout the day, with values ranging from 18 to 21 °C and 10–14 °C, respectively, when using the single and dual PM systems. The large temperature gap observed between the single (18 °C) and triple (4 °C) systems represents a decisive difference in product safety, directly linked to substantial reductions in bacterial growth rates. Overall, the figures confirm that cooling performance is strongly dependent on both environmental conditions and module count, with the triple-module setup achieving the only temperature suitable for fish preservation. These results are consistent with the findings of some of the following studies: Where^41^, perform temperature control in the range of 5 to 25 ᵒC, and provide cooling. Also^42^, concluded that the PM cooling system powered by solar energy could lead to a qualitative leap in industrial cooling systems by providing a sustainable, cost-effective, and eco-friendly solution. As well^14^, maintain the fish’s freshness with the reference temperature of 0–5 ᵒC by using a cool box based on solar photovoltaic, thermoelectric cooler (TEC). Furthermore^43^, investigated cooling using PM to cool water from 40 ᵒC to 16 ᵒC. Additionally^44^, fabricated a solar thermoelectric cooler (STC) to cool fish at 5 ᵒC.

**Fig. 8:**
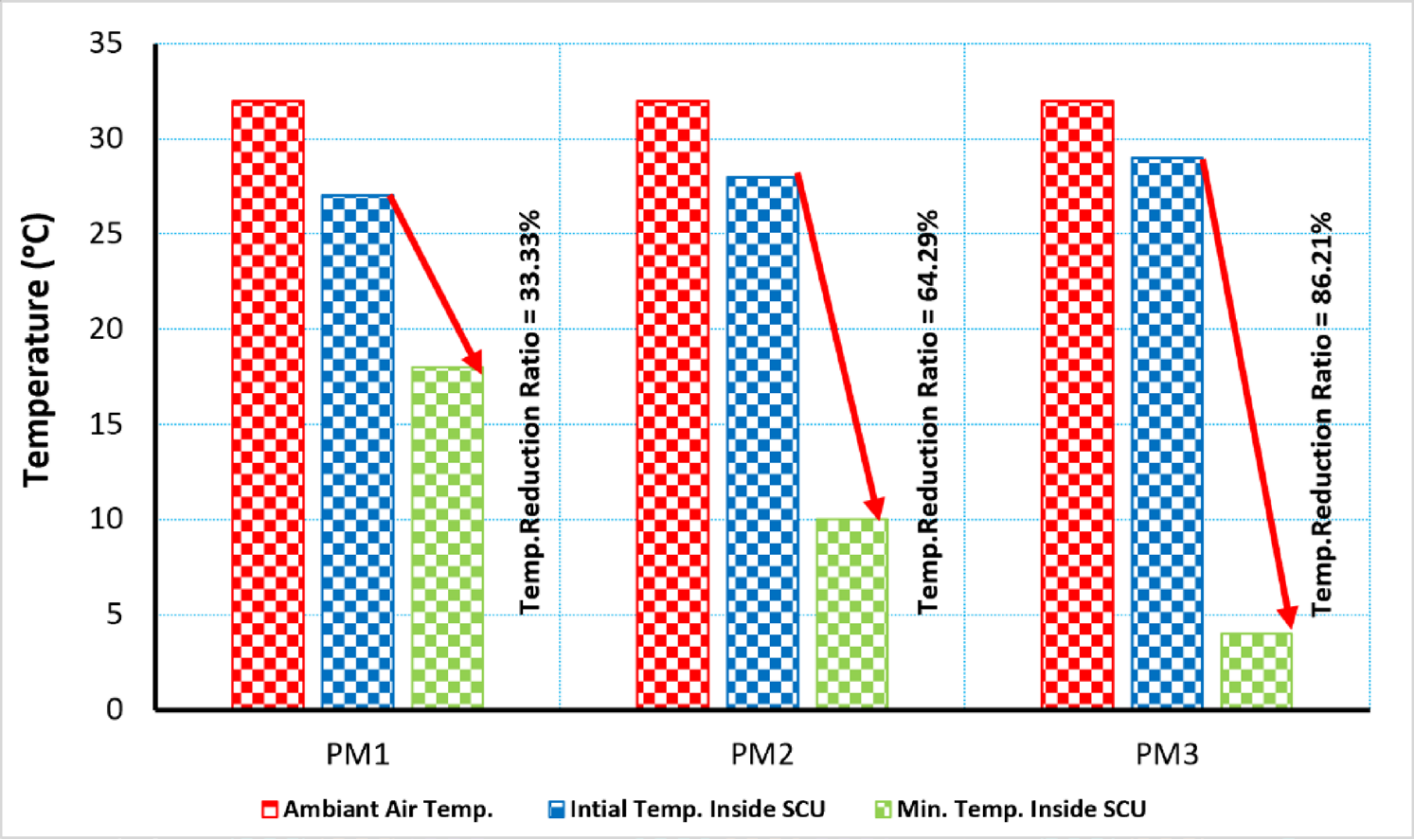
Illustrates the temperature reduction ratio of the SCU at different PM.

**Fig. 9:**
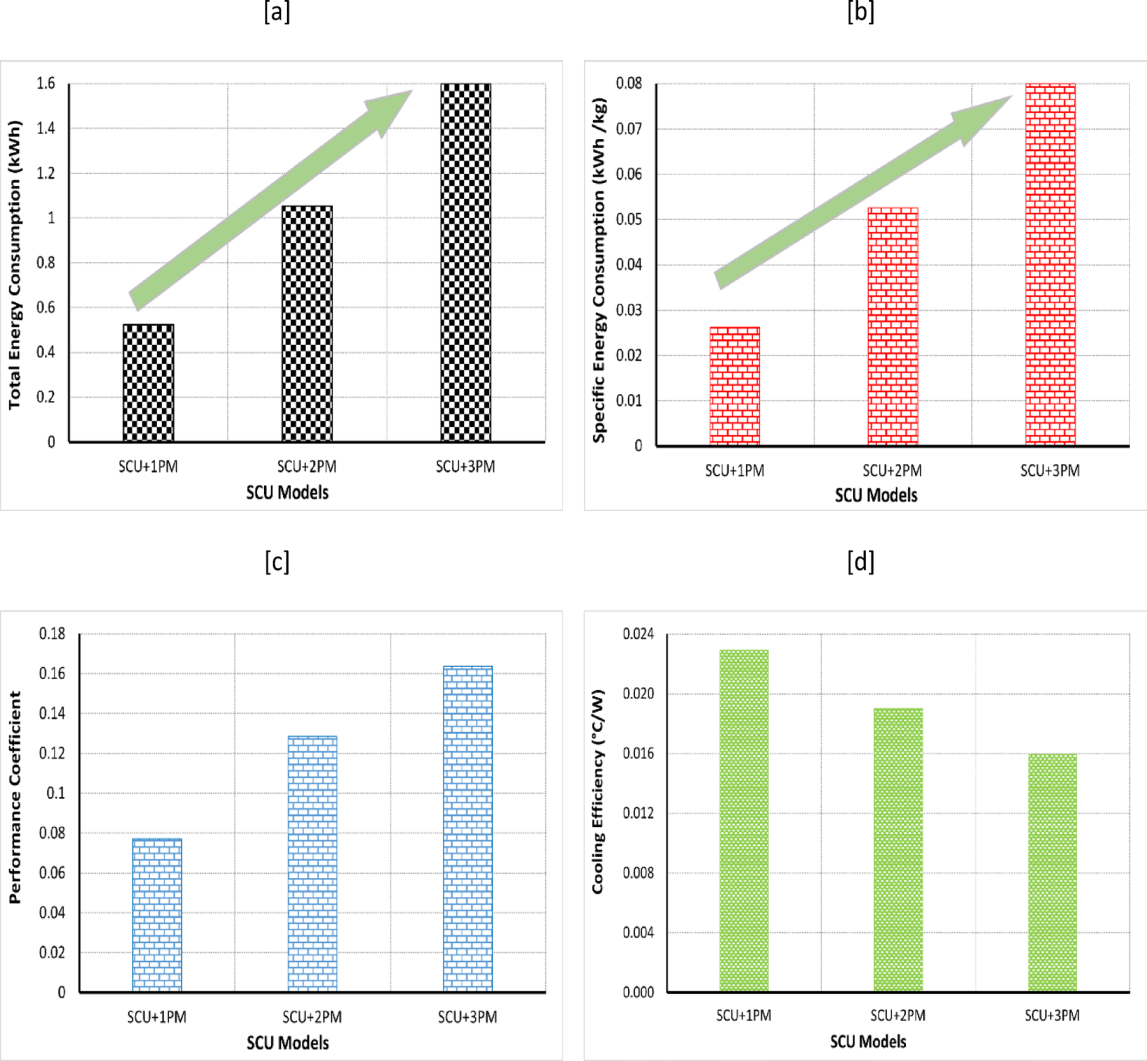
Shows the evaluation criteria for the SCU. [**a**] total energy consumption, [**b**] specific energy consumption, [**c**] performance coefficient, [**d**] cooling efficiency.

In this context, Fig. 8 highlights the percentage decrease in temperature inside the SCU at the initial cooling time point and the stability of the ambient air inside the cooling chamber at the lowest cooling temperature compared to the ambient air temperature in the operating environment. The lowest reduction ratio in temperature inside the cooling chamber, at a value of 86.21%, was clearly observed in the triple PM configuration cooling system. Simultaneously, the lowest percentage decrease in temperature within the cooling chamber environment was recorded in the single and dual PM systems, at 33.33% and 64.29%, respectively. This is attributed to the reduction in the number of PM systems from the three PM units to the single- and double PM unit systems. In the context of evaluating the solar cooling unit, the energy consumption results showed an upward linear pattern, increasing with the number of PM (single, dual, and triple PM configurations), confirming predictable energy budgeting across system designs. Figure 9**[a]** shows the findings of the total energy consumption of the SCU, where the triple PM units attached to the SCU achieved the highest total energy consumption with a value of 1.6 kWh. Then came the second and third phases, solar cooling units attached to dual and single PM units with values of 1.1 and 0.5 kWh.

Similarly, the results for specific energy consumption (SEC) were recorded, with the triple PM units attached to the SCU registering the highest specific energy consumption at a value of 0.079 kWh/kg, followed by values of 0.052 and 0.026 kWh/kg for the dual and the single PM units attached to the SCU, as shown in Fig. 9**[b]**. The behavior of energy consumption results is generally due to the power requirements for operating the PM, as the more PMs there are inside the SCU, the greater the power requirements for operation. The results showed that all values remained extremely low - well below 0.1 kWh/kg - and significantly more efficient than conventional cooling technologies. Overall, the combined interpretation of the four panels demonstrates a critical engineering conclusion: despite the increase in power demand, only the triple-module configuration offers preservation-grade performance. The higher energy use is fully justified by its ability to achieve and maintain the critical 4 °C storage temperature. Conversely, increasing the cooling capacity of the SCU came at the expense of its thermodynamic efficiency, as illustrated in Fig. 9**[c]**. The triple PM cooling system recorded the lowest performance cooling coefficient with a value of 0.039, followed by the dual and single PM cooling systems with values of 0.046 and 0.056. This may be due to the increased electricity consumption in units that include more than one Peltier unit. It is noteworthy that this system has a lower COP compared to conventional vapor compression systems, which range from 2 to 4. However, this system offers the advantage of using conventional energy, relying on solar power, and reducing greenhouse gas emissions due to solar energy use. Furthermore, its lower cost makes it suitable for small-scale fishermen. The behavior of the cooling performance lab results, in terms of their decrease with increasing cooling time when using different cooling systems, is consistent with both^46,62–64^. It should be noted that the reported COP values represent the overall system-level coefficient of performance rather than the intrinsic thermoelectric module COP reported in TEC manufacturer datasheets. The calculation incorporates the total electrical consumption of the complete cooling unit, including Peltier modules, fans, and pump operation, under actual field conditions characterized by high ambient temperatures and unavoidable thermal gains. Consequently, the effective system COP is expected to be lower than the ideal module COP reported under controlled laboratory conditions. Following the same behavior as the performance coefficient results for the SCU, the cooling efficiency is shown in Fig. 9**[d]**. The single PM cooling system recorded the highest cooling efficiency with a value of 0.018 °C/W, followed by the dual and triple PM cooling systems with values of 0.014 and 0.012 °C/W.

### Uncertainty indicators

Regarding the expanded measurement uncertainty for all measured quantities across the three solar-powered TEM cooling configurations, single PM, dual PM, and triple PM were presented in Table 4. The analysis follows the internationally recognized Guide to the Expression of Uncertainty in Measurement^51^, and incorporates both repeatability (Type A) and instrument accuracy (Type B) components, as recommended by^65^. The ambient air temperature (T_air), relative humidity (RH), and solar irradiance (SR) are common environmental conditions shared across all three configurations, as measurements were conducted simultaneously. Their uncertainty values are therefore reported once. The cooling chamber temperature (T_cool) and temperature difference (ΔT = T_air − T_cool) are configuration-dependent and are reported separately for each case. For the Triple PM configuration, the mean T_cool reached 5.91 °C with an expanded uncertainty of ± 1.88 °C, confirming with 95% confidence that the true cooling temperature lies between 4.03 °C and 7.79 °C. This range satisfies the^54^ fish preservation standard of T_cool ≤ 7 °C, providing strong experimental evidence for the system’s suitability for fish storage applications. The Type A component (repeatability) was derived from 11 repeated steady-state hourly readings (08:00–18:00, excluding t = 0 at system startup), capturing the natural variability of each measured quantity under real operating conditions. The Type B component was obtained from the manufacturer’s instrument specifications. For the K-type thermocouple (**IEC 60584-1 Class 1**), the manufacturer’s accuracy is ± 1.5 °C, yielding u_B = 0.87 °C - the dominant source of uncertainty in T_cool measurements. This is consistent with the general finding that instrument accuracy is the primary uncertainty contributor in thermoelectric system experiments.

**Table 4:** Expanded measurement uncertainty for the three peltier module configurations.

Configuration	Parameter	Unit	*n*	Mean	σ	u_A (Type A)	u_B °C (Type B)	u_c (Combined)	U (k = 2) (Expanded)
Environmental Parameters	T_air	°C	11	32.52	2.124	0.64	0.46	0.79	± 1.58
	RH	% RH	11	48.17	7.52	2.27	1.73	2.85	± 5.71
	SR	Wm^− 2^	11	615.9	255.3	76.97	5.77	77.19	± 154.4
Single PM (1 × 60 W)	T_cool	°C	11	19.36	1.027	0.31	0.87	0.92	± 1.84
	ΔT	°C	—	13.16	—	—	—	1.21	± 2.42
Dual PM (2 × 60 W)	T_cool	°C	11	11.82	1.250	0.38	0.87	0.95	± 1.89
	ΔT	°C	—	20.70	—	—	—	1.23	± 2.46
Triple PM (3 × 60 W)	T_cool	°C	11	5.91	1.221	0.37	0.87	0.94	± 1.88
	ΔT	°C	—	26.61	—	—	—	1.23	± 2.46

(GUM, JCGM 100:2008; Coverage factor k = 2; Confidence level ≈ 95%)

PM: is the pelter module; n: is the number of readings; σ: is the standard deviation.

The uncertainty in ΔT was obtained by error propagation, as ΔT is a derived quantity rather than a directly measured one. For the K-type thermocouple (**IEC 60584-1 Class 1**), the manufacturer’s accuracy is ± 1.5 °C, yielding u_B = 0.87 °C — the dominant source of uncertainty in T_cool measurements. This is consistent with the general finding that instrument accuracy is the primary uncertainty contributor in thermoelectric system experiments. The uncertainty in ΔT was obtained by error propagation, as ΔT is a derived quantity rather than a directly measured one. The combined uncertainty u_c (ΔT) was calculated using the law of propagation of uncertainty, which accounts for the correlated contributions of T_air and T_cool uncertainties.

### Monte carlo statistical parameters analysis

**Fig. 10:**
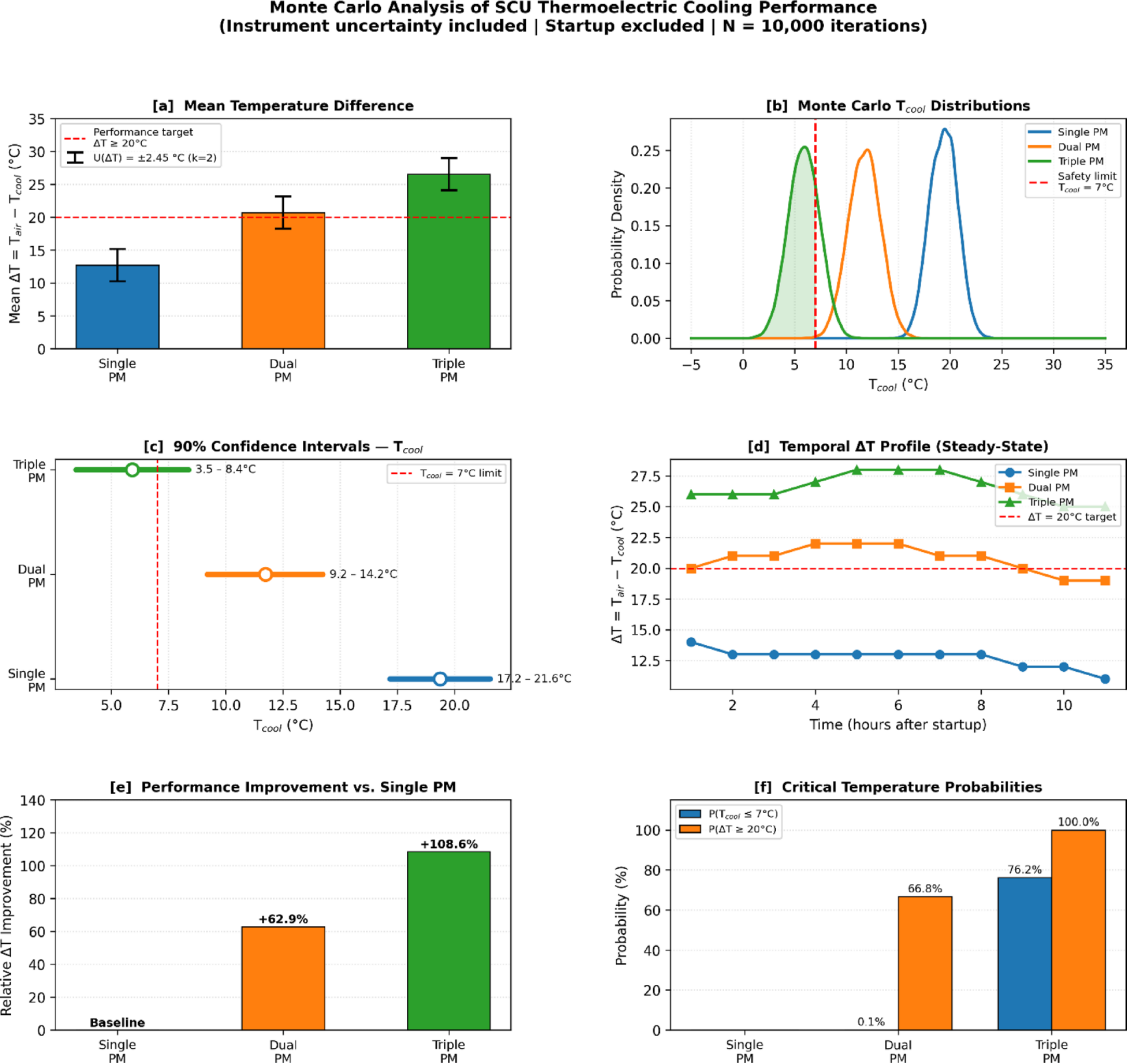
Illustrates the Monte Carlo statistical analysis. [**a**] T_cool_ comparison, [**b**] ΔT distributions, [**c**] 90% confidence intervals, [**d**] Δt time series, [**e**] Performance improvement, [**f**] Critical temperature probabilities.

To assess the durability and reliability of the SCU cooling performance under realistic operating variability, a Monte Carlo simulation was performed based on the experimentally measured cooling chamber temperature T_cool and temperature difference ΔT = T_air − T_cool, for the single, dual, and triple Peltier configurations. The steady-state statistical parameters derived from the experimental measurements (*n* = 11 per configuration, startup transient excluded) were: Single PM (µ = 19.36 °C, σ = 1.03 °C), Dual PM (µ = 11.73 °C, σ = 1.27 °C), and Triple PM (µ = 5.91 °C, σ = 1.22 °C). These parameters defined the Normal probability distributions from which Monte Carlo samples were drawn. The analysis considered six key evaluation parameters: (i) ΔT comparison, (ii) Monte Carlo probability distributions, (iii) 90% confidence intervals, (iv) temporal behavior of ΔT, (v) relative performance improvement, and (vi) critical temperature probabilities, as summarized in Fig. 10. Figure 10**[a]** shows a clear enhancement in cooling performance with increasing module count: the SCU with a triple-PM configuration achieved the highest mean temperature difference inside the cooling chamber (ΔT = 26.55 °C), corresponding to a 108.6% improvement compared to the single-PM configuration (ΔT = 12.73 °C).

The dual-PM configuration exhibited an intermediate mean ΔT of 20.73 °C. The error bars (± 1 SD) indicate consistent behavior within each configuration, and the absence of overlap between the cooling performance ranges confirms that the three configurations are statistically distinct in terms of cooling capability. These findings regarding the temperature drop inside the SCU are consistent with previous studies on thermoelectric and solar-powered cooling systems. The probability density functions derived from the Monte Carlo simulations in Fig. 10**[b]** exhibit well-defined normal distributions for T_cool in each configuration. The triple-PM distribution is centered at the lowest T_cool values (µ = 5.91 °C), with a significant portion falling below the 7 °C fish-preservation threshold, indicating a robust separation in cooling performance rather than random experimental scatter. The relatively narrow distribution widths reflect acceptable variability, while the systematic leftward shift of the distributions with increasing module count confirms predictable and scalable performance. The Fig. 10**[c]** further enhances this differentiation by showing the 90% confidence intervals for T_cool. The estimated confidence ranges are 17.2–21.6 °C, 9.2–14.2 °C, and 3.5–8.4 °C for the single, dual, and triple configurations, respectively, with no overlap between them, demonstrating that the observed performance differences are unlikely to result from random variability. Notably, the triple-PM confidence interval (3.5–8.4 °C) straddles the 7 °C preservation threshold, confirming that its cooling performance is in the critical fish-preservation range. The temporal evolution of ΔT throughout the daylight period is presented in Fig. 10**[d]**. After an initial transient phase, all configurations reach a quasi-steady behavior, with the triple-PM SCU maintaining ΔT ≥ 20 °C over most of the operating window, whereas the single-PM configuration remains below 13 °C for the entire test duration. This stable behavior highlights the operational reliability of multi-module configurations for continuous cooling applications in field-like conditions. The relative cooling performance improvement with respect to the single-PM baseline is quantified in Fig. 10**[e]**. The dual and triple configurations provide 62.9% and 108.6% higher mean ΔT, respectively, indicating a near-linear performance scaling where each additional module contributes approximately 50–60% incremental improvement, which is particularly useful for practical system sizing. Finally, Fig. 10**[f]** reports the critical temperature probabilities, which constitute the main added value of the Monte Carlo framework compared with simple descriptive statistics. The triple-PM configuration achieves a 76.2% probability of achieving T_cool ≤ 7 °C [54, 55], i.e., it reliably meets the fish-preservation threshold under the tested variability, while the single and dual configurations exhibit significantly lower probabilities of reaching this target. Thus, the Monte Carlo analysis not only confirms the experimental trends, but also provides reliability-oriented metrics (confidence intervals and failure/success probabilities) that quantify the risk of under-performance and support robust design and module-number selection for small-scale fishers.

While a 76.2% probability of achieving T_cool_ ≤ 7 °C implies a nearly 1 in 4 chance of temperature excursions above the ideal threshold, this represents a transformative improvement for artisanal fishermen who previously relied on ambient storage, where quality degradation was highly prevalent. Brief temperature excursions (e.g., reaching 7.5 °C) during active fishing hours do not result in immediate spoilage but rather slightly accelerate the loss of premium freshness. To push this reliability metric above the 90% threshold for future commercialization, several thermal optimization strategies can be employed. These include upgrading the chamber insulation from standard plywood to injected Polyurethane (PU) foam to minimize conduction, integrating Phase Change Materials (PCMs) within the chamber walls to act as thermal buffers against frequent door openings, and employing water-cooled heat sinks to maximize heat rejection efficiency on the hot side of the Peltier modules.

### Cost analysis

Regarding the cost analysis of the SCU for preserving fish during the fishing operation at sea, Table 5 demonstrates the results of the cost analysis parameters of the SCU.

**Table 5:** Shows the findings of the SCU cost analysis.

Cost Analysis Parameters	Unit	SCU ($)	SCU (LE)
Principal cost of the SCU structure (*PC*_*SCU*_)	$	182	8668.04
Capital recovery factor (CRF)		0.15	0.15
First annual cost of the SCU ($$\:{Cost}_{SCU}$$)	$	26.57	1265.53
Lifespan average (n) (year)	year	15	15
Interest rate in (IR)	%	12	12
Salvage value	$	36.4	1733.61
Salvage annual	$	0.98	46.81
Sinking fund factor (SFF)		0.027	0.027
Annual maintenance cost of the SCU (*MC*_*annual*_)	$	27.3	1300.21
Total annual cost of the SCU	$	52.89	2518.97
Average annual productivity (Avg. Productivity)	kg	720	720
cost of chilled fish/ kilogram produced (CCF_kg_)	$	0.074	3.52
Price of yield/kg	$	2	95.25
Payback period (year)	year	0.15	0.15

The annual cost of chilled fish/ kilogram produced (CCF_kg_) of the SCU was valued at 0.1 $ ≈ 3.52 LE, and the payback period of the SCU WAS 0.15 years. The cost analysis parameters presented that the utilization of the SCU attached with a triple PM cooling system reduced the payback period. These economic feasibility study indicators suggest that the SCU with a triple PM cooling system is the most economically advantageous, enhancing the shortest payback period.

## Conclusions

A Clear methodology to optimize thermally of the SCU for preserving fish during fishing has been presented in this work based on utilizing solar energy. In addition, a general computational model, using Monte Carlo statistical analysis, simulates the behavior of any cooling system under any circumstances, focusing on the influence of different factors, such as the ΔT comparison, Monte Carlo distributions, confidence intervals, temporal behavior, relative performance improvement, and critical temperature probabilities. As well, an economic analysis also focused on the feasibility of the solar cooling model and the payback period. Despite some limitations of the study, such as conducting tests only in August, Egypt enjoys ample sunshine throughout the year, which encourages wider adoption of the technology. While the study used only one fish species to test the technology, this is a good indicator of its potential application to a wider range of fish species. The findings indicated that the lowest temperature reduction inside the SCU, 4 ᵒC, was achieved using a triple PM configuration at the highest specific energy consumption with a value of 0.099 kWh/kg, and a minimum energy consumption coefficient with a value of 0.036 kWh/kg. The single PM cooling system recorded the highest performance cooling coefficient with a value of 0.055, followed by the dual and triple PM cooling systems with values of 0.043 and 0.036. The Monte Carlo statistical analysis showed that the relative cooling performance improvement values were 67 and 108% for dual and triple PM, compared to the single PM. These findings were further supported by the results of the feasibility study and payback period for the SCU. The SCU, incorporating three Peltier elements, achieved a low payback period of 0.15 years. This facilitates the dissemination of this technology, which is suitable for small-scale fishermen and protects the environment by reducing greenhouse gas emissions due to its reliance on clean solar energy. Developing and testing this unit under varying loads, with multiple fish species, and under different operating conditions (both summer and winter), along with a comparative study of most fish cooling systems, a detailed study will also conducted to estimate greenhouse gas emissions resulting from the use of different fish cooling systems, are among the most important recommendations of this study for future investigation and deployment of this technology to a diverse range of stakeholders.

## Data Availability

All data are provided within the paper.
